# Spontaneous Renal Artery Pseudoaneurysm Causing Subcapsular Haematoma in the Setting of Acute Pyelonephritis: A Rare Clinical Nexus and Review of the Literature

**DOI:** 10.7759/cureus.111323

**Published:** 2026-06-22

**Authors:** Deelip Kumar Singh, Ankur Mittal, Vikas K Panwar, Aayush Lakkanna, Abinash Paul

**Affiliations:** 1 Urology, All India Institute of Medical Sciences, Rishikesh, Rishikesh, IND

**Keywords:** bacterial pyelonephritis, endovascular coil embolisation, mycotic pseudoaneurysm, renal artery pseudoaneurysm, spontaneous subcapsular renal hematoma, superselective embolisation, transarterial embolisation, type 2 diabetes mellitus, wunderlich syndrome

## Abstract

Spontaneous renal subcapsular haematoma caused by a renal artery pseudoaneurysm (RAP) arising in the context of acute pyelonephritis is an exceptionally rare event. This combination, infective arteriopathy leading to pseudoaneurysm formation with subcapsular haemorrhage, has seldom been reported and represents a clinically challenging diagnostic and therapeutic scenario.

A 42-year-old woman with poorly controlled type 2 diabetes mellitus presented with right flank pain, fever and acute-on-chronic kidney disease. Imaging revealed bilateral pyelonephritis, a progressively enlarging right renal subcapsular haematoma (9.7 × 9.6 cm on computed tomography {CT} angiography) and a pseudoaneurysm arising from the inferior segmental branch of the right renal artery. She underwent successful selective right renal artery coil embolisation with subsequent clinical and biochemical improvement, without requiring nephrectomy.

Infectious arteriopathy in the setting of acute pyelonephritis, particularly in immunocompromised diabetic patients, may culminate in mycotic pseudoaneurysm formation and spontaneous subcapsular haemorrhage. Early CT angiography and prompt endovascular intervention are pivotal to preserving the kidney and patient survival.

## Introduction

Spontaneous, non-traumatic subcapsular or perirenal renal haemorrhage, historically designated as Wunderlich syndrome after the German physician Carl August Wunderlich, who first described it in 1856, is a rare urological emergency [1]. The syndrome classically manifests as Lenk's triad: acute flank pain, a palpable flank mass and signs of hypovolemic shock, though this complete triad is present in fewer than 25% of cases [2]. Contemporary literature estimates the incidence of renal angiomyolipoma at 0.07%-0.3% [3].

The aetiology is dominated by renal neoplasms, which account for approximately 61% of cases in the landmark meta-analysis by Zhang et al. of 165 cases; angiomyolipoma and renal cell carcinoma are the most common benign and malignant causes, respectively [4]. Vascular pathology, including polyarteritis nodosa, renal artery aneurysms and pseudoaneurysms, constitutes the next most frequent category at approximately 17%-25%. Infectious aetiology, including pyelonephritis, accounts for only 2%-12% of cases and represents the rarest clinical subset [4,5].

Renal artery pseudoaneurysm (RAP) is a contained vascular injury in which the adventitia or perivascular connective tissue, rather than true arterial wall, forms the aneurysm wall. While RAP most commonly arises following renal biopsy, percutaneous nephrolithotomy (PCNL), partial nephrectomy or blunt trauma, mycotic pseudoaneurysms arising from infective arteritis or proteolytic vessel-wall degradation during active infection are distinctly uncommon [6,7]. A particularly rare subset involves pseudoaneurysm formation secondary to pyelonephritis in a native kidney, with only isolated case reports in the literature [7,8].

We present the case of a 42-year-old diabetic woman in whom acute bilateral pyelonephritis was complicated by a right renal artery pseudoaneurysm arising from the posterior segmental branch, causing a progressively enlarging subcapsular haematoma, successfully treated by selective transarterial coil embolisation with the preservation of renal function. We review the relevant published literature and discuss the pathophysiology, diagnostic approach and management strategy for this rare clinical entity.

## Case presentation

Clinical history and presentation

A 42-year-old woman, a known case of type 2 diabetes mellitus for 15 years (on irregular medications {tablet metformin 500 mg twice daily}, HbA1c: 14.1%), presented to the emergency department of All India Institute of Medical Sciences (AIIMS), Rishikesh, with a four-day history of right flank pain that had progressively generalised to all four quadrants, a single febrile episode 10 days prior (resolved spontaneously), two episodes of non-bilious vomiting and a one-day history of decreased urine output and rapidly progressive dyspnoea (modified Medical Research Council {mMRC} grades III-IV). There was no history of trauma, urological instrumentation or previous renal intervention.

On examination, the patient was conscious and oriented and vitally stable. Pallor was present. Right renal angle tenderness was elicited on percussion. Bilateral basal crepitations were noted on respiratory auscultation, more pronounced on the right. No pedal oedema was present.

Investigations

Haematological and biochemical investigations are shown in Table 1.

**Table 1:** Laboratory parameters PT, prothrombin time; INR, international normalised ratio; CRP, C-reactive protein

Investigation	Value	Reference Range
Haemoglobin	7.0 g/dL	12.0-15.5 g/dL
Total leucocyte count	7,500/μL	4,000-11,000/μL
Platelet count	1.51 × 10⁵/μL	1.50-4.00 × 10⁵/μL
Serum creatinine (day 1 of admission)	6.73 mg/dL	0.5-1.1 mg/dL
Serum creatinine (day 2 of admission)	7.55 mg/dL	0.5-1.1 mg/dL
Blood urea	250 mg/dL	15-40 mg/dL
PT	16.7 seconds	11.0-13.5 seconds
INR	1.49	0.8-1.2
CRP (day 1 of admission)	266.4 mg/L	<6.0 mg/L
CRP (day 5 of admission)	196 mg/L	<6.0 mg/L
HbA1c	14.1%	<5.7%-6.4%
Urine culture (day 1 of admission)	No growth	No growth
Blood culture	No growth	No growth

Imaging

Non-contrast computed tomography scan of the kidneys, ureters and bladder (CT KUB) (Figure 1) demonstrated gross right renal enlargement (14.3 × 7.4 cm) with a hyperdense subcapsular collection (~58 Hounsfield units {HU}) of maximum thickness of 1.8 × 6.4 cm along the posterior, lateral and inferior aspects, extending into the pararenal space, consistent with subcapsular haematoma. Bilateral perirenal fat stranding with pararenal conal fascial thickening was present. Contrast-enhanced computed tomography (CECT) scan was not performed in view of impaired renal function (estimated glomerular filtration rate {eGFR}: ~15 mL/minute/1.73 m²).

**Figure 1:**
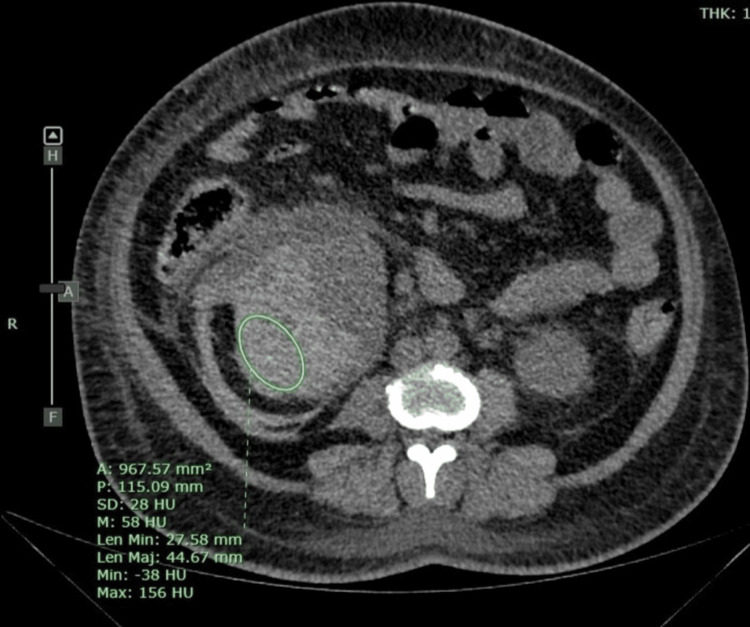
Axial non-contrast CT KUB section shows a subscapular hyperdense collection in the lower pole of the right kidney CT KUB: computed tomography scan of the kidneys, ureters and bladder

In view of the spontaneous subcapsular haematoma without a traumatic or iatrogenic aetiology, abdominal CT angiography (CTA) was performed on day 5 of admission after a second session of haemodialysis had partially improved renal function. This demonstrated a small saccular outpouching within the right renal subcapsular collection, showing attenuation equivalent to the aorta on all phases, consistent with a pseudoaneurysm arising from an inferior segmental branch of the right renal artery (Figure 2). By this time, the haematoma had enlarged to 9.7 × 9.6 cm. The left kidney showed perirenal fat stranding without a collection. No other visceral aneurysm was identified.

**Figure 2:**
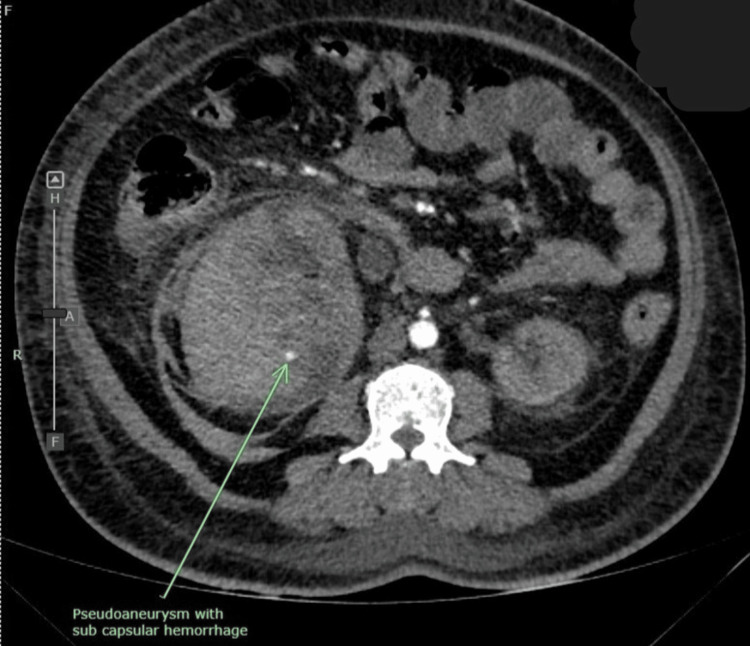
Axial CT angiography of the abdominal section showing a hyperdense lesion in the lower pole of the right kidney consistent with a pseudoaneurysm and a large subscapular haematoma along the lower pole CT: computed tomography

Management and outcome

The selective catheterisation of the right renal artery was performed, and angiography confirmed the pseudoaneurysm arising from the inferior segmental branch (Figure 3). The feeding artery was selectively catheterised, and the pseudoaneurysm was embolised with microcoils (Figures 4, 5). Post-embolisation angiography demonstrated contrast stasis confined to the pseudoaneurysm sac, confirming technical success. Post-procedure was uneventful.

**Figure 3:**
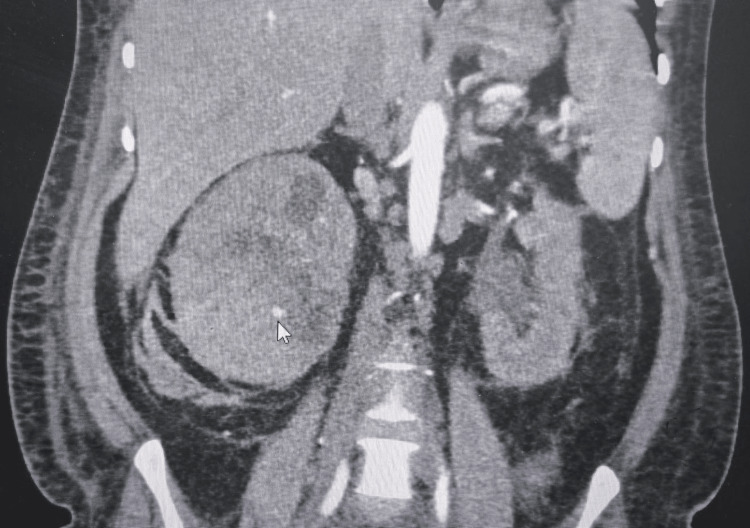
Coronal CT angiography of the abdominal section showing a hyperdense lesion in the lower pole of the right kidney consistent with a pseudoaneurysm and a large subscapular haematoma along the lower pole CT: computed tomography

**Figure 4:**
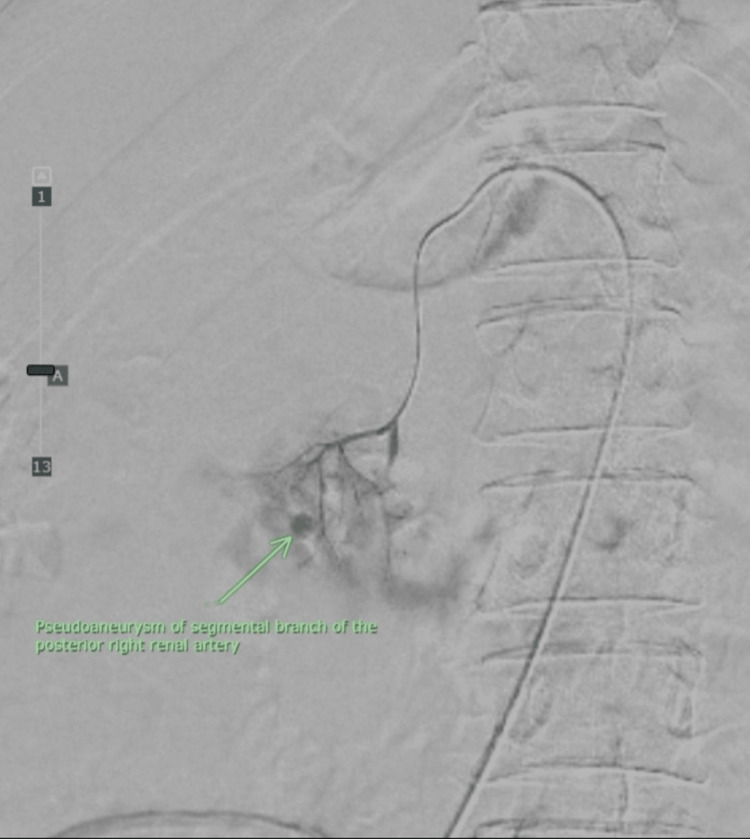
Digital subtraction angiography arrow showing the pseudoaneurysm of the inferior segmental branch of the right renal artery

**Figure 5:**
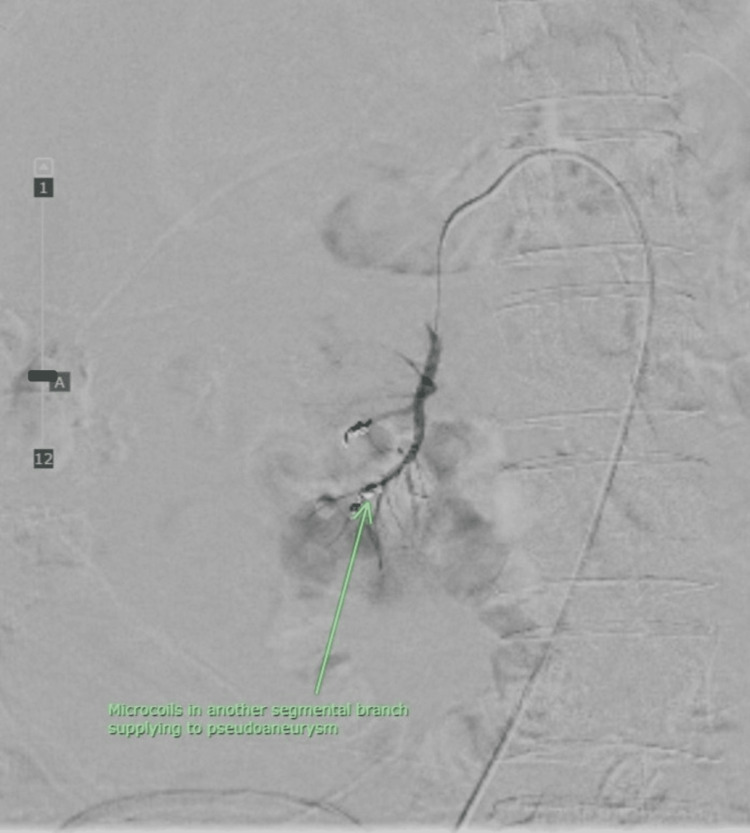
Digital subtraction angiography arrow showing the angioembolisation of the pseudoaneurysm of the inferior segmental branch of the right renal artery with microcoils

Concurrently, the patient was managed for sepsis secondary to bilateral pyelonephritis with intravenous (IV) antibiotics on clinical grounds. Two sessions of haemodialysis were required for refractory hyperkalaemia and high anion gap metabolic acidosis; no further dialysis was needed after day 6, indicating partial renal recovery. Laboratory parameters were gradually improved. Severely deranged glycaemia was managed with IV insulin.

## Discussion

This case is notable for three concurring exceptional features: first, spontaneous renal subcapsular haematoma in the context of acute pyelonephritis without a neoplastic aetiology, an event reported in fewer than 2.4% of Wunderlich syndrome cases [4]; second, the identification of a mycotic segmental renal artery pseudoaneurysm as the proximate vascular source of haemorrhage, a complication rarely documented in the setting of native-kidney pyelonephritis [7,8]; and third, successful nephron-preserving endovascular management in a patient with pre-existing chronic kidney disease and active sepsis.

Wunderlich syndrome: Epidemiology and aetiology

Among infectious aetiologies, pyelonephritis, including the xanthogranulomatous variant, renal abscess and emphysematous forms, has been documented as a precipitant in sporadic case reports. Itagaki et al. (2024) described an 80-year-old diabetic woman with *Klebsiella pneumoniae* pyelonephritis causing left subcapsular haematoma (Wunderlich syndrome), who required surgical drainage despite antibiotic therapy [9]. García-Chairez et al. (2022) reported a 38-year-old diabetic woman with bilateral pyelonephritis and right renal abscess in whom an acute haemodynamic deterioration necessitated emergency nephrectomy [5]. Subtil et al. (2024) reported a 54-year-old man with *K. pneumoniae* bacteraemia and pyelonephritis complicated by a 9.1 × 7.9 × 2.8 cm subcapsular haematoma, managed conservatively [1]. Critically, none of these cases, nor the series reviewed by Gupta et al. (2025) of pyelonephritis-associated spontaneous perinephric haematoma, demonstrated a concomitant renal artery pseudoaneurysm as the vascular source [10]. The co-occurrence of infective pseudoaneurysm causing subcapsular haematoma in the index case thus represents a unique and more complex clinical phenotype.

Pathophysiology of infective pseudoaneurysm in pyelonephritis

Mycotic (infective) renal artery pseudoaneurysms arise through distinct mechanisms differing fundamentally from traumatic or iatrogenic counterparts. In the context of pyelonephritis, the predominant pathogenic mechanisms include (i) direct bacterial invasion and proteolytic degradation of the arterial wall by virulent Gram-negative organisms secreting collagenase, elastase and hyaluronidase; (ii) infective arteritis driven by the haematogenous seeding of the vasa vasorum; and (iii) extrinsic arterial compression and ischaemic weakening by adjacent microabscesses or inflammatory phlegmon [6,11]. Koyama et al. (2024) reported the most analogous published case: a 53-year-old man with calculous pyelonephritis in whom a ruptured renal pseudoaneurysm developed on day 8 of hospitalisation, treated with successful angioembolisation; the authors postulated that bacterial proteolytic enzymes from *Klebsiella pneumoniae* caused direct arterial wall injury [8].

The role of severely uncontrolled diabetes mellitus in the index patient warrants specific emphasis. Hyperglycaemia impairs leucocyte chemotaxis, phagocytosis and intracellular bacterial killing, facilitating the bacterial invasion of vessel walls while simultaneously promoting endothelial dysfunction and heightened susceptibility of the arterial media to proteolytic degradation [11]. The HbA1c of 14.1% in the index patient represents extreme metabolic derangement. Furthermore, diabetic vascular disease renders small- and medium-sized renal vessels more vulnerable to infective arteriopathy. The negative blood cultures and echocardiography findings effectively excluded endocarditis and haematogenous embolisation as alternative mechanisms, supporting a direct infective arteritis model in this case.

Diagnostic approach

Non-contrast CT (NCCT) was appropriately selected as the initial modality in this patient with an eGFR of 15 mL/minute/1.73 m². On NCCT, a hyperdense collection (≥35-65 HU) in the subcapsular or perirenal space is diagnostic of acute haematoma [2]. However, NCCT cannot characterise the vascular aetiology or identify a pseudoaneurysm. The decisive investigation was abdominal CT angiography, which, by demonstrating a saccular outpouching with attenuation equivalent to the aorta on all phases, confirmed a pseudoaneurysm with high specificity. CTA has become the imaging modality of choice for suspected renal vascular injury, offering sensitivity approaching that of digital subtraction angiography (DSA) with the added advantage of the three-dimensional anatomical mapping for endovascular planning [6].

An important clinical lesson from this case is the absence of gross haematuria, a classic symptom of renal pseudoaneurysm, likely because the pseudoaneurysm ruptured into the subcapsular space rather than the collecting system. This underscores that any patient with pyelonephritis who fails to improve clinically despite appropriate antibiotics, or who develops unexplained or worsening anaemia with subcapsular collection on imaging, should undergo urgent cross-sectional imaging to exclude a vascular source [1,9].

Endovascular management

Selective renal artery embolisation (SRAE) is established as the first-line intervention for haemodynamically stable renal pseudoaneurysm, with technical success rates exceeding 90% in published series [6,12]. The nephron-preserving advantage of selective embolisation over surgical nephrectomy is particularly important in patients with pre-existing CKD, as in the index case. The coil embolisation of the feeding segmental branch achieves thrombosis of the pseudoaneurysm sac while preserving flow in adjacent branches, thereby limiting ischaemic parenchymal loss [12]. Ohebshalom et al. (2001) first demonstrated the feasibility of endovascular embolisation for mycotic renal pseudoaneurysm, in the context of bacterial endocarditis, as a haemostatic bridge to definitive surgery [11]. The present case extends this approach to the setting of native-kidney pyelonephritis-related pseudoaneurysm, with definitive cure achieved by embolisation alone, without subsequent nephrectomy.

Post-embolisation angiography confirming contrast stasis within the pseudoaneurysm sac with preserved renal artery branching represents the standard criterion for technical success, as was achieved in the index case.

Unique aspects of this case and clinical lessons

To our knowledge, this case represents one of the rare documented instances of a spontaneous infective renal artery pseudoaneurysm arising from a named segmental branch, confirmed on CT angiography, causing progressive subcapsular haematoma in the setting of acute pyelonephritis, without antecedent trauma, instrumentation, biopsy or endocarditis. The successful nephron-preserving endovascular management with subsequent partial renal recovery (creatinine improvement from 7.55 mg/dL to 3.77 mg/dL) in a patient who required dialysis at presentation is a particularly encouraging outcome.

Limitations

Several limitations of this report merit acknowledgement. First, as a single-centre case report, this case documents an isolated patient and cannot support or generalise conclusions regarding the incidence, natural history or optimal management of pyelonephritis-associated renal artery pseudoaneurysm. The rarity of this clinical entity precludes formal epidemiological analysis from a single case.

Second, urine and blood cultures were sterile on two occasions, precluding the microbiological confirmation of the causative organism. While the clinical, biochemical and radiological context strongly favours an infective aetiology, particularly given the active bilateral pyelonephritis and severe diabetic immunocompromise, an underlying subclinical vasculopathy or occult connective tissue disorder could not be formally excluded in the absence of the histopathological sampling of the aneurysm wall or the microbiological identification of the causative pathogen.

Third, the contrast-enhanced CT (CECT) of the abdomen was deferred at initial presentation due to severely impaired renal function (eGFR: ~15 mL/minute/1.73 m²), meaning that the pseudoaneurysm was not identified until day 5 after two sessions of haemodialysis. An earlier diagnosis might have been possible with the use of an alternative modality such as contrast-enhanced ultrasound (CEUS), which carries no nephrotoxic risk; however, CEUS availability and operator expertise remain limited in many resource-constrained settings, including ours.

Fourth, long-term follow-up data beyond the immediate post-embolisation period are not available. The durability of the embolisation, subsequent renal function trajectory, the risk of recurrent pseudoaneurysm and the long-term fate of the subcapsular haematoma could not be assessed from the current follow-up interval. Extended surveillance with imaging and renal function monitoring is planned but not yet reported.

## Conclusions

Spontaneous renal subcapsular haematoma secondary to an infective renal artery pseudoaneurysm in the setting of acute pyelonephritis is a rare but potentially life-threatening complication, particularly in immunocompromised patients with poorly controlled diabetes mellitus.

The unique severity of this case, pseudoaneurysm-driven haematoma expansion in the context of bilateral pyelonephritis, acute-on-chronic kidney disease requiring dialysis and extreme glycaemic dysregulation, highlights the multifactorial vulnerability of the diabetic patient to this rare but serious vascular complication. Unexplained anaemia, failure to respond to antibiotics or incidentally detected subcapsular collection in any patient with pyelonephritis should prompt urgent CT angiography to exclude a vascular source. When a pseudoaneurysm is identified, selective transarterial coil embolisation offers a safe, effective and nephron-preserving therapeutic option. Multidisciplinary collaboration is essential to optimise outcomes in these complex patients.
